# Tipping the balance between good and evil: aberrant 14-3-3ζ expression drives oncogenic TGF-β signaling in metastatic breast cancers

**DOI:** 10.1186/s13058-015-0603-2

**Published:** 2015-07-11

**Authors:** Chevaun D. Morrison, William P. Schiemann

**Affiliations:** Case Comprehensive Cancer Center, Case Western Reserve University, Wolstein Research Building, 2103 Cornell Road, Cleveland, OH 44106 USA

## Abstract

Transforming growth factor beta (TGF-β) readily suppresses the development of early-stage breast cancers, an activity that gives way to tumor promotion in their late-stage counterparts. The molecular mechanisms underlying this mysterious switch in TGF-β function remain murky. In addressing this conundrum, Xu et al. observed aberrant 14-3-3ζ expression to prevent the formation of tumor-suppressive Smad2/3:p53 complexes, while simultaneously driving the generation of oncogenic Smad2/3:Gli2 complexes. Once formed, Smad2/3:Gli2 complexes stimulate the expression of parathyroid hormone-related protein necessary for breast cancer metastasis to bone. This viewpoint highlights 14-3-3ζ as an essential driver of oncogenic signaling by Smad2/3 and TGF-β in metastatic breast cancers.

## Background

Transforming growth factor beta (TGF-β) is a ubiquitously expressed cytokine that plays essential roles in regulating tissue development, differentiation, and homeostasis [1, 2]. TGF-β also functions as a prominent suppressor of mammary tumorigenesis, doing so by inhibiting mammary epithelial cell (MEC) proliferation and inducing their apoptosis [1, 2]. The diverse cellular responses and anti-tumor activities of TGF-β transpire in part through its “canonical” activation of the latent transcription factors Smad2 and Smad3, which interact with the transcriptional machinery to manage the transcriptome in a cell-specific and context-specific manner [3]. Interestingly, while early-stage breast cancers remain reliant upon canonical TGF-β signaling and its tumor-suppressive activities, the progression and eventual metastasis of their late-stage counterparts depends upon the activation of noncanonical (i.e., Smad2/3-independent) TGF-β effectors, including mitogen-activated protein (MAP) kinases, phosphoinositide 3-kinase (PI3K)/AKT, nuclear factor (NF)-κB, and Yes-associated protein (YAP)/transcriptional co-activator with PDZ-binding motif (TAZ) [4, 5]. The paradoxical switch in TGF-β function during metastatic progression provided the impetus to develop targeted chemotherapies designed to inactivate the TGF-β pathway and its oncogenic activities in late-stage carcinomas [6, 7]. Unfortunately, anti-TGF-β agents have exhibited limited success in clinical settings owing to an incomplete understanding of “how, when, and why” TGF-β acquires oncogenic activity in metastatic settings. Equally perplexing is the extent to which the canonical and noncanonical TGF-β signaling systems contribute to metastatic progression. Thus, overcoming these knowledge gaps and defining the contextual clues whereby Smad2/3 signals coalesce with noncanonical effectors in driving oncogenic TGF-β signaling will be essential to improving the efficacy of anti-TGF-β therapies.

## The article

The 14-3-3 family of adapter molecules is comprised of seven members (i.e., 14-3-3β, 14-3-3γ, 14-3-3ε, 14-3-3ν, 14-3-3σ, 14-3-3τ, and 14-3-3ζ) that can function to either suppress or promote tumorigenesis, doing so by forming homodimeric and heterodimeric complexes that bind and sequester more than 200 phosphoproteins [8, 9]. For instance, 14-3-3σ functions as a potent tumor suppressor through its ability to regulate p53 expression, an event frequently inactivated in breast cancers due to epigenetic silencing of 14-3-3σ expression [10, 11]. In stark contrast, aberrant 14-3-3ζ expression is readily observed in several human malignancies and predicts for poor prognoses in patients harboring breast, lung, or head and neck cancers [12]. Along these lines, elevated 14-3-3ζ expression couples TGF-β to the initiation of epithelial–mesenchymal transition (EMT) and metastatic programs in mammary tumors [13], suggesting that dysregulated 14-3-3ζ expression and activity may play an essential role in regulating oncogenic TGF-β signaling.

**Fig. 1:**
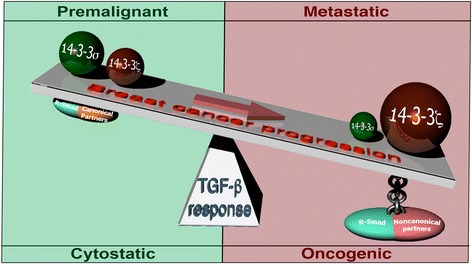
Tipping the balance in transforming growth factor beta (TGF-β) signaling in metastatic breast cancers. Expression of 14-3-3σ coordinates cytostatic TGF-β signals in premalignant MECs, while 14-3-3ζ expression and activity drives the acquisition of oncogenic TGF-β signaling in metastatic breast cancer cells. See text for additional details

Indeed, in a provocative study published in *Cancer Cell*, Xu et al. [14] established 14-3-3ζ as a “molecular switch” that converts TGF-β from acting as a tumor suppressor to a tumor promoter in metastatic breast cancers (Fig. 1). In doing so, the authors showed that the enforced expression of 14-3-3ζ inactivates the tumor-suppressing functions of 14-3-3σ, particularly its ability to stabilize p53 and coordinate the formation of Smad2/3:p53 complexes operant in driving p21 expression. Likewise, 14-3-3ζ binds and sequesters phosphorylated YAP1 within the cytoplasm, thereby preventing its nuclear translocation and transactivation of 14-3-3σ expression. Collectively, these events coalesce to inactivate cytostatic TGF-β signaling in premalignant MECs. Interestingly, rendering bone tropic breast cancer cells deficient in 14-3-3ζ expression dramatically reduced their metastatic colonization and secondary outgrowth in the bones of mice. This cellular condition also prevented metastatic breast cancer cells from activating osteoclast maturation and bone osteolysis in response to TGF-β. Indeed, TGF-β-mediated bone metastasis required 14-3-3ζ to stabilize Gli2 expression, leading to the formation of Smad2/3:Gli2 complexes that drive parathyroid hormone-related protein (PHrT) expression. These findings are consistent with 14-3-3ζ expression being embedded in TGF-β gene signatures coupled to breast cancer metastasis to bone, an event that significantly reduces bone metastasis-free survival in breast cancer patients. Collectively, this intriguing study establishes 14-3-3ζ as a preemptive molecule operant in inactivating the cytostatic functions of TGF-β in premalignant MECs, and as an essential mediator that unifies canonical and noncanonical TGF-β signaling outputs during breast cancer metastasis to bone.

## Viewpoint

Overexpression of 14-3-3ζ has been observed in numerous human cancers, including those of the esophagus, breast, lung, brain, and prostate [12, 15]. More recently, 14-3-3ζ has been detected in the secretomes produced by these cancers, suggesting that monitoring 14-3-3ζ expression may serve as a potential prognostic biomarker for late-stage breast carcinomas [9, 14]. Equally intriguing is the finding by Xu et al. that 14-3-3ζ is capable of dictating the expression patterns of additional 14-3-3 family members (e.g., 14-3-3σ), thereby generating an extensive level of control capable of fine-tuning the context and pathological output of metastatic signals stimulated by TGF-β. Accordingly, Boudreau et al. [16] demonstrated that 14-3-3σ, which typically functions as a tumor suppressor, may in fact enhance the invasive behaviors of basal-like breast cancer cells, doing so by regulating cytoskeletal dynamics in a protein kinase Cζ-dependent manner. In both studies, aberrant 14-3-3 family member activity was associated with basal-like and triple-negative breast cancers (TNBCs), both of which respond to the tumor-promoting activities of TGF-β. It should be noted that basal-like/TNBCs are generally not highly metastatic to bone and instead preferentially metastasize to visceral organs and the brain [17], which contrasts sharply with their estrogen receptor alpha-positive counterparts that do in fact preferentially metastasize to bone and are responsive to the tumor-suppressing activities of TGF-β [18, 19]. Future studies need to: (1) identify which specific basal-like/TNBC subtypes are dependent upon dysregulated 14-3-3 family and oncogenic TGF-β signaling; (2) establish the impact of aberrant 14-3-3 family member expression in non-TNBC subtypes, as well as their response to TGF-β; and (3) demonstrate a causal role of dysregulated 14-3-3 family expression in dictating breast cancer dissemination to organ sites other than bone.

Finally, it is interesting to note that the activities associated with aberrant 14-3-3ζ expression are highly reminiscent of those attributed to the oncogenic functions of TGF-β, including the ability to drive carcinoma cell proliferation, survival, and chemoresistance [1, 2, 9]. The highlighted work by Xu et al. implicates 14-3-3ζ as a prominent player that dictates the pathophysiology of canonical TGF-β signaling in neoplastic cells. However, TGF-β also activates numerous noncanonical signaling effectors that are equally essential in mediating the tumor-promoting functions of TGF-β in developing and progressing breast cancers [4]. Future studies thus clearly need to expand our understanding of the role of 14-3-3 family members in eliciting metastatic progression driven by TGF-β, particularly with respect to determining the extent to which (1) aberrant 14-3-3ζ expression engages the noncanonical TGF-β signaling system, (2) noncanonical TGF-β effectors engender dysregulated expression of 14-3-3ζ, and (3) aberrant 14-3-3ζ expression can be targeted therapeutically and/or utilized as a predictive biomarker to delineate breast cancer patients most likely to respond to anti-TGF-β agents. Ultimately, answering these and other questions will provide a foundation to develop more effective therapies against the oncogenic functions of TGF-β and its stimulation of breast cancer metastasis.

## Abbreviations

EMT: Epithelial–mesenchymal transition
MAP: Mitogen-activated protein
MEC: Mammary epithelial cell
NF: Nuclear factor
PHrT: Parathyroid hormone-related protein
PI3K: Phosphoinositide 3-kinase
TAZ: Transcriptional co-activator with PDZ-binding motif
TGF-β: Transforming growth factor beta
TNBC: Triple-negative breast cancer
YAP: Yes-associated protein
